# Concept Mapping Plays a Complementary Role in Optimizing the Effectiveness of Interactive Simulations in Medical Student Learning of Bacterial Sepsis Pathophysiology

**DOI:** 10.1007/s40670-025-02348-1

**Published:** 2025-03-07

**Authors:** Blaine Traylor, Emma Fenner, Adam Western, Brendan Seabold, Allison Mool, Jacob Schmid, Thomas Johnston, Dajonea Robinson, Akhila Kambhatla, Pruthvi Sainath Reddy, William Thomas, Tobias Merriman, Pickney Benedict, Shelley Tischkau, Donald Torry, Gabriel J. Tobón, Richard Selinfreund

**Affiliations:** 1 Mayo Clinic Hospital, Phoenix, AZ USA; 2 University of Tennessee, Memphis, TN USA; 3 Department of Medical Microbiology, Immunology and Cell Biology, Southern Illinois University School of Medicine, 825 N. Rutledge St. Room 2639, Springfield, IL 62794-9626 USA; 4 Center for Virtual Expression, Southern Illinois University, Carbondale, IL USA; 5 School of Computing, Southern Illinois University, Carbondale, IL USA; 6 Computer Science, University of Illinois, Springfield, IL USA; 7 Department of Medical Education, Southern Illinois University School of Medicine, 825 N. Rutledge St., Room 2639, Springfield, IL 62794-9626 USA; 8 Anthropology, Southern Illinois University, Carbondale, IL, USA

**Keywords:** Concept mapping, 3D computer simulation, Bacterial sepsis, Medical education

## Abstract

The increasing complexity of medical education requires innovative tools to help students manage cognitive overload. Concept mapping (CM) enhances knowledge retention and integration by visually organizing information, and has received positive feedback in various educational contexts. However, the use of CM combined with interactive 3D courseware to teach the cellular mechanisms of bacterial sepsis is underexplored. This study evaluated the feasibility and effectiveness of integrating a 3D computer simulation with CM to improve understanding of sepsis. Fifty-two second-year medical students were randomized into three groups (A, B, and C). Each group completed five tasks: a pre-simulation self-assessment, a scaffolded pre-simulation CM, a computer simulation, a post-simulation CM, and a post-simulation self-assessment. Group A completed the tasks at the start of the sepsis case in the medical curriculum, Group B in the middle, and Group C at the end. A control group completed only the pre-simulation CM, and third-year students who had previously participated repeated the CM. Pre-simulation CM scores showed significant improvement in all three groups compared to the control group (*p* < 0.05). Post-simulation, Group C significantly outperformed Groups A and B (*p* < 0.05). Third-year students scored 15 points higher than Groups A and B (*p* < 0.001), but their scores were similar to Group C’s. CM scores improved across all intervention groups, with Group C showing more than double the increase seen in Groups A and B. This study suggests that combining 3D simulations with CM is an effective strategy for teaching complex medical concepts and warrants further exploration of its long-term impact.

## Introduction

The growing complexity of medical education presents significant challenges for educators, students, and curriculum developers. With the rapid expansion of biomedical knowledge and the increasing cognitive demands placed on learners, traditional teaching methods, such as lectures and text resources, may no longer be sufficient to ensure deep integration and retention of essential medical concepts [1–4]. Stakeholders in medical education—including faculty, administrators, and students—must navigate these challenges by identifying effective instructional strategies that enhance comprehension, critical thinking, and clinical reasoning. In particular, mastering multifaceted conditions such as bacterial sepsis requires students to synthesize molecular mechanisms with clinical applications, a task that can be overwhelming without structured learning tools [5, 6]. As the leading cause of in-hospital mortality worldwide, sepsis is a life-threatening condition that medical students must understand thoroughly, from its molecular underpinnings to clinical manifestations [7, 8]. To address these concerns, innovative approaches that promote active learning, such as concept mapping (CM) and interactive 3D simulations, offer promising solutions.

CM is an established educational tool designed to enhance knowledge organization, integration, and retention. According to Novak and Gowin [9], CMs are schematic tools for representing relationships between key concepts in a structured, hierarchical manner. By visually organizing information, CM allows learners to make explicit connections between ideas, fostering critical thinking and deeper comprehension [10]. This technique has been widely applied in medical education to promote active learning, improve clinical reasoning, and support decision-making skills. CM serves as a valuable method for structuring and representing knowledge, enabling learners to consolidate and interpret their ideas while making their thought processes more visible. By promoting an organized approach to learning, CM contributes to a more profound comprehension of complex subjects. Its effectiveness as an educational tool has been demonstrated across multiple disciplines, and its application in medical education continues to expand, offering promising benefits for student learning [11].

CM has proven to be effective for evaluating critical thinking skills in medical education [12, 13]. Additionally, CM can be used as both a formative and a summative assessment tool, allowing educators to assess students’ understanding and monitor knowledge development over time [14–19]. When it comes to providing feedback, CM helps students clarify and refine their understanding of a subject. Educators, in turn, can use these maps as a tool to offer structured and meaningful feedback, facilitating deeper learning [20]. Furthermore, CM plays a significant role in programmatic assessment strategies [21], where multiple data points are used to guide assessment decisions, ensuring a comprehensive evaluation of student progress. Students can create CM before and after a lecture series or course, providing instructors with insights into their cognitive structure and understanding of the subject matter. This approach enables educators to assess how well students integrate and organize knowledge over time.

One of the primary challenges in utilizing CM for assessment lies in the scoring methodology. Two predominant scoring approaches have emerged: (1) structural scoring, based on the framework developed by Novak and Gowin [9], which evaluates hierarchical structuring, the use of linking words, cross-links, and examples; and (2) relational scoring, which focuses on the number and accuracy of propositions and the overall coherence of the map [22, 23]. In the relational scoring method, the strength and relevance of conceptual connections play a central role in determining the quality of the map, focusing not only on the number of links but also on the depth of meaning they convey. In addition, CM can be used in both lecture courses and clinical experiences to support learning. However, despite its established benefits, the integration of CM with interactive 3D courseware, particularly in teaching the intricate cellular mechanisms underlying diseases like bacterial sepsis, remains an underexplored area.

The advent of new computer-based learning technologies presents an opportunity to strengthen the integration of basic science and clinical medicine in medical curricula. Serious games have increasingly been utilized as an educational approach, actively engaging learners and enhancing motivation and satisfaction [24], adapted to support clinical education and decision-making [1, 25, 26]. Simulation-based learning is a well-established method in medical education [25–27]. Effective strategies are continuously being developed to translate traditional medical simulations into virtual environments. These visual simulations incorporate images, animations, videos, and 3D environments to enhance the learning experience. Computer simulations that integrate decision-making elements based on molecular disease mechanisms and link them to potential patient outcomes offer a valuable tool for reinforcing these connections.

In the current study, we developed a 3D computer-simulated environment designed to support medical students’ understanding of the cellular mechanisms leading to bacterial sepsis. This interactive platform incorporates gamification elements, such as case-based challenges and decision-making tasks, to engage learners in applying their knowledge to clinical scenarios. The simulation aims to provide a dynamic environment where students explore questions in real time. To further enhance student engagement, the simulation includes estimates of cost, mortality, and length of hospital stay as key game metrics.

Combining CM with 3D simulation offers a novel, dual-faceted approach to medical education. The interactive simulation provides an intuitive, visual representation of cellular mechanisms, while CM reinforces conceptual linkages and cognitive organization. This synergistic integration has the potential to extend students’ understanding of sepsis by allowing them to construct, refine, and interconnect core concepts dynamically.

To address these educational challenges, this study introduces a 3D computer-simulated environment combined with CM into the medical school curriculum. We aim to assess the feasibility, utility, and student perceptions of this combined approach in enhancing understanding of the cellular processes that lead to bacterial sepsis. By evaluating CM performance and student engagement with the simulation, we seek to determine whether this innovative educational strategy can improve medical students’ knowledge acquisition, retention, and application in clinical practice.

## Materials and Methods

### Study Design and Participants

**Fig. 1:**
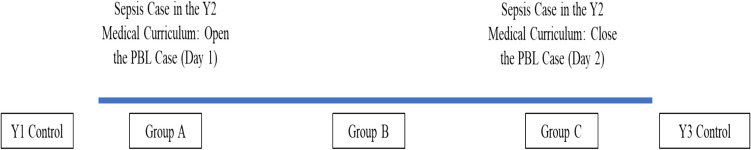
Timeline of activities related to the Y2 medical curriculum. In our medical curriculum, the Problem-Based Learning (PBL) case opens and closes within the same week. On Day 1 (**opening**), students are presented with a clinical vignette that describes a patient’s chief complaint, relevant medical history, and initial physical examination findings. The group collaborates to identify key problems and knowledge gaps. They hypothesize possible diagnoses, discuss relevant pathophysiology, and brainstorm the additional information needed, such as diagnostic tests (e.g., blood work, imaging). Based on this discussion, the group creates learning objectives to investigate further. Over the next couple of days, each student reviews the learning objectives independently. On Day 2 (**closing**), students present and discuss the research they completed, addressing the learning objectives and applying their new knowledge to the case. They refine their differential diagnoses, and additional clinical information, including diagnostic test results or the patient’s clinical progress, is provided. This allows the group to reach a final diagnosis and discuss treatment options. In our study, **Group A** completed the tasks at the start of the sepsis case (on the same day the case opened), **Group B** completed them midway through the case, and **Group C** completed them at the end (after the case had closed). First-year students (with a foundation in immunology) completed only the pre-simulation concept map (CM), while third-year students who had previously participated repeated the CM

The research team recruited second-year medical students as volunteers from the infectious disease and immunology unit. A total of 52 students were randomly assigned to three groups based on their last names (Fig. 1): Group A (*n* = 17), Group B (*n* = 18), and Group C (*n* = 17). Additionally, a control group (*n* = 15) consisted of Year 1 medical students who completed only the pre-simulation CM without exposure to the sepsis case, and a third-year student cohort (*n* = 10) who had completed the full activity the previous year was included to assess knowledge retention.

### Procedures and Intervention

All students in the intervention groups completed five sequential tasks:

1. **Pre-simulation self-assessment survey**: Students rated their knowledge of the cellular mechanisms of sepsis prior to the simulation.
2. **Pre-simulation scaffolded CM**: Students created a CM outlining the cellular basis of bacterial sepsis before engaging with the simulation.
3. **Interactive 3D courseware** (**computer simulation)**: The simulation platform illustrated the cellular and hemodynamic processes of bacterial sepsis, emphasizing immune responses and the effects of antimicrobial therapy on patient outcomes.
4. **Post-simulation scaffolded CM**: After completing the simulation, students revised or recreated their CM to reflect their updated understanding.
5. **Post-simulation self-assessment survey**: Students reassessed their knowledge of the case material after the simulation.

Group A completed these tasks at the beginning of the sepsis case in the medical curriculum, Group B at the midpoint, and Group C at the end. The control group only completed the pre-simulation CM and did not participate in the simulation. The third-year students completed the CM at the end of the case but did not engage with the simulation a second time.

The surveys aimed to assess students’ self-perceived knowledge gaps in immunology, microbiology, and pathology while evaluating the impact of a computer simulation on their awareness of these gaps. To achieve this, a pre-simulation survey was administered, followed by an almost identical post-simulation survey. The survey consisted of 17 items designed to measure students’ stress levels, anxiety related to academic situations, confidence in understanding and studying key scientific concepts, and their ability to make clinical connections in medicine. Most questions used a 5-point Likert scale, ranging from strong disagreement to strong agreement or from none to maximum (1 = strongly disagree; 5 = strongly agree). Additionally, the survey included a question on students’ attitudes toward gamified educational materials and an open-ended response for comments or suggestions. In the post-simulation survey, students rated their readiness and knowledge of disease processes related to sepsis and provided feedback on the simulation. This structured approach captured both quantitative and qualitative data to evaluate baseline perceptions and the educational intervention’s effectiveness.

### Interactive 3D Courseware (Computer Simulation)

**Fig. 2:**
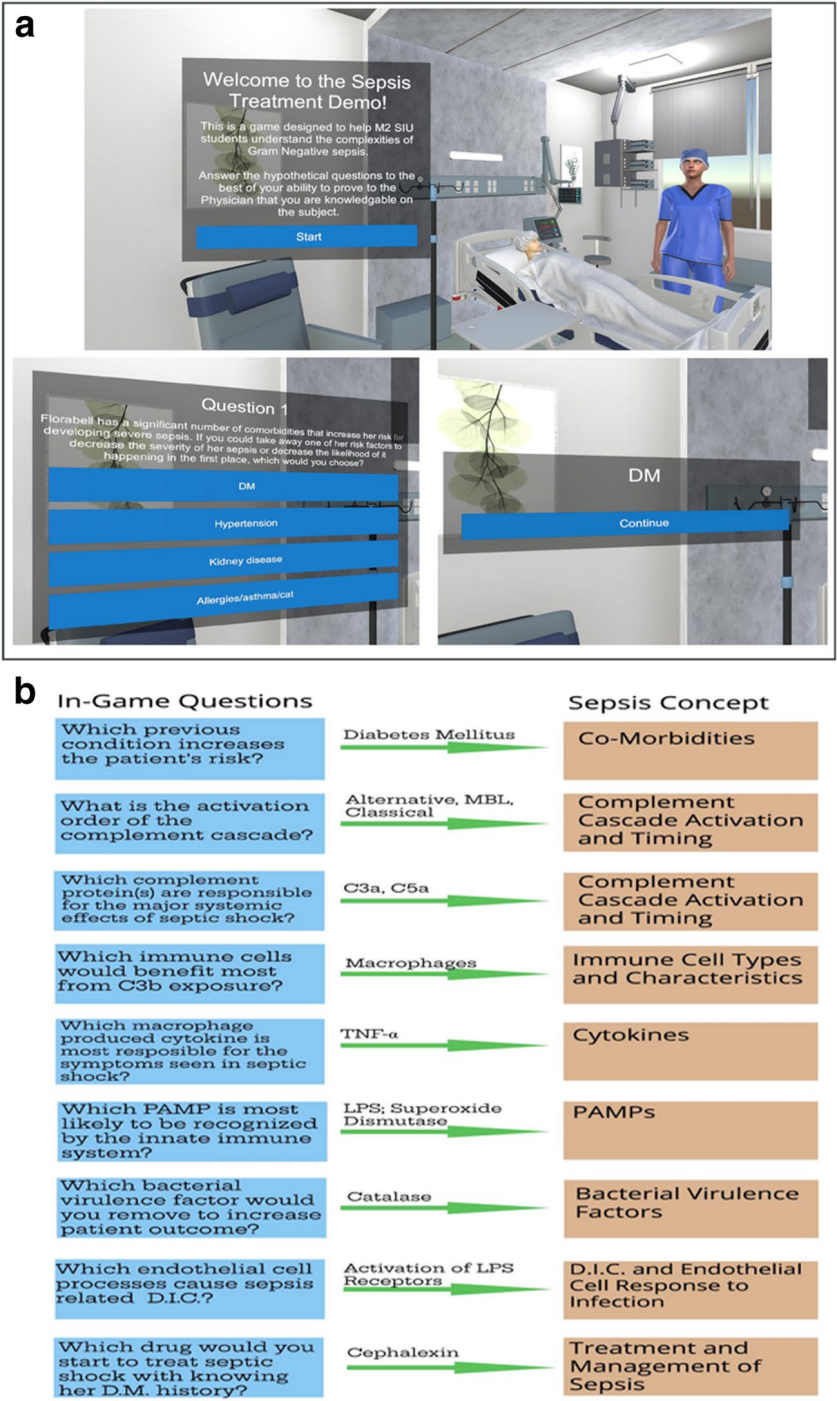
**a** Screenshots from the gamification of sepsis simulation illustrating co-morbidities related to sepsis. **b** Decision-making point questions provided to the learners in the computer simulation

A 3D simulation of gram-negative sepsis was developed for this study to connect the knowledge of cellular mechanisms involved in bacterial sepsis with simulated patient outcomes. The simulation begins with an introductory case presentation, providing a visual context that sets the stage for a series of questions and decisions that the player must navigate. The game illustrates the cellular steps involved in the progression of the disease, linked to a patient health bar (Fig. 2A). The focus of the simulation is on enhancing critical decision-making skills, recognizing key symptoms and biomarkers, and understanding the cellular cascade of events that occur during the various phases of sepsis. Decision-making points were strategically designed as questions to highlight essential cellular events in the disease progression, emphasizing the importance of timely disease recognition (Fig. 2B). To further enhance student engagement, the simulation includes estimates of cost, mortality, and length of hospital stay as key game metrics. Learners were provided a section at the end of the post-computer session questionnaire to provide feedback on the computer simulation.

### Medical Simulation Development Platform

For this simulation, we employed Unity software, a game development engine that enabled us to create an immersive and visually engaging learning environment. The simulation begins with a brief, informative video that introduces a patient displaying symptoms indicative of sepsis. Following this introduction, learners encounter a series of questions and decision points that focus on the cellular interactions contributing to the cascade of immunological events leading to disease progression. The decision points are structured to maintain a realistic sequence of events. The questions and decision points emphasize the critical cellular steps involved in disease progression, allowing learners to understand the impact of their choices in a safe, controlled academic environment. Correct answers move the health bar indicator into the green range, while incorrect answers shift the indicator toward the red range. Each student completed the computer simulation in the medical library, where they also filled out surveys and created CMs in a single, uninterrupted session. On average, the total time required to complete the surveys, concept maps, and the computer simulation was approximately 45 min.

### Concept Map Activity

The CM activity was developed locally by a dedicated team of educators with expertise in medical education, immunology, and clinical reasoning. Its primary objective was to assess the integrative and applied learning of medical students, reflecting the complexity, interconnectivity, and progressive nature of medical knowledge. The CM activity was designed to mirror the cognitive processes involved in clinical reasoning, offering an opportunity for students to demonstrate their ability to organize, apply, and synthesize key concepts within clinical contexts.

#### Initial Concept Provision

To initiate the CM activity, students were provided with a curated set of core concepts central to the topic (a map with empty concepts). These concepts, selected by the research team for their potential to branch into multiple related areas, included foundational ideas such as “Pathogenicity & Virulence,” “Cellular Immune Response,” and “Comorbidities.” These core principles served as anchors for students’ CM, promoting a balance between structured thinking and analytical freedom. The goal was for students to organize the information around these key ideas while allowing them to explore and uncover additional connections and relationships, mirroring the depth and complexity of real-world clinical situations.

#### Scaffolded Framework

The CM was scaffolded to reflect the clinical reasoning process, in which differential diagnoses are systematically narrowed down based on emerging information. Categories like “Comorbidities,” “Cellular Immune Response,” and “Pathogenicity & Virulence” were pre-established to provide a structured framework. The “Treatment” category was emphasized as the central clinical outcome, guiding students to connect mechanistic understanding with clinical action. This scaffolded approach ensured that students considered both foundational science and clinical application, enhancing their ability to integrate theoretical knowledge with clinical decision-making.

#### Hierarchy and Cross-linking

A key feature of the CM was the emphasis on hierarchy and cross-links, which are essential for effective clinical reasoning. The hierarchy of concepts followed the progression from basic scientific principles to clinical application, symbolizing the depth and layered structure of medical knowledge. Students were encouraged to draw cross-links between distinct topics, simulating how clinicians integrate information from various domains when addressing complex patient cases. These cross-links reflected the interdisciplinary nature of medical practice and provided insight into how clinicians connect seemingly unrelated areas of knowledge to form a comprehensive understanding of a patient’s condition.

#### Scoring Rubric Development

The CM scoring rubric was developed locally by the research team, informed by best practices in CM assessment. The scoring criteria placed a premium on the quality and depth of student understanding, prioritizing higher-order thinking, synthesis, and application over surface-level recall. The rubric was structured around three key components:

- **Hierarchy:** Students were assessed on the logical arrangement of concepts within the map. Higher points were awarded for appropriately organized primary, secondary, and tertiary levels of information that demonstrated a clear progression from basic concepts to clinical outcomes.
- **Cross-links:** Points were given for identifying meaningful, non-trivial connections between different areas of knowledge. Cross-links signified a nuanced understanding of the material and reflected the students’ ability to integrate various concepts and disciplines.
- **Relationships:** The accuracy and specificity of relationships between concepts were evaluated, with additional points awarded for students who could clearly elucidate the underlying mechanisms or clinical correlations driving the connections. This criterion assessed the depth and precision of students’ understanding.

To ensure objectivity in scoring, each CM was evaluated by an instructor with expertise in both the subject matter and CM assessment. The instructor was blinded to the students’ group assignments (pre- or post-simulation) and identities to eliminate potential bias. Performance on the CM was compared across groups: students who completed the CM before and after the simulation, as well as a control group and third-year medical students who had previously participated in similar activities.

#### Validity and Design Considerations

Although this CM approach did not undergo formal validity testing, it was carefully designed with input from both medical educators and researchers, ensuring it adhered to established best practices in concept mapping. The scaffolded map was crafted to provide structure while allowing sufficient flexibility for students to engage in higher-order thinking. The evaluation rubric, focusing on hierarchical organization, cross-linking, and relationship depth, was designed to emphasize the integration and application of knowledge rather than rote memorization.

**Fig. 3:**
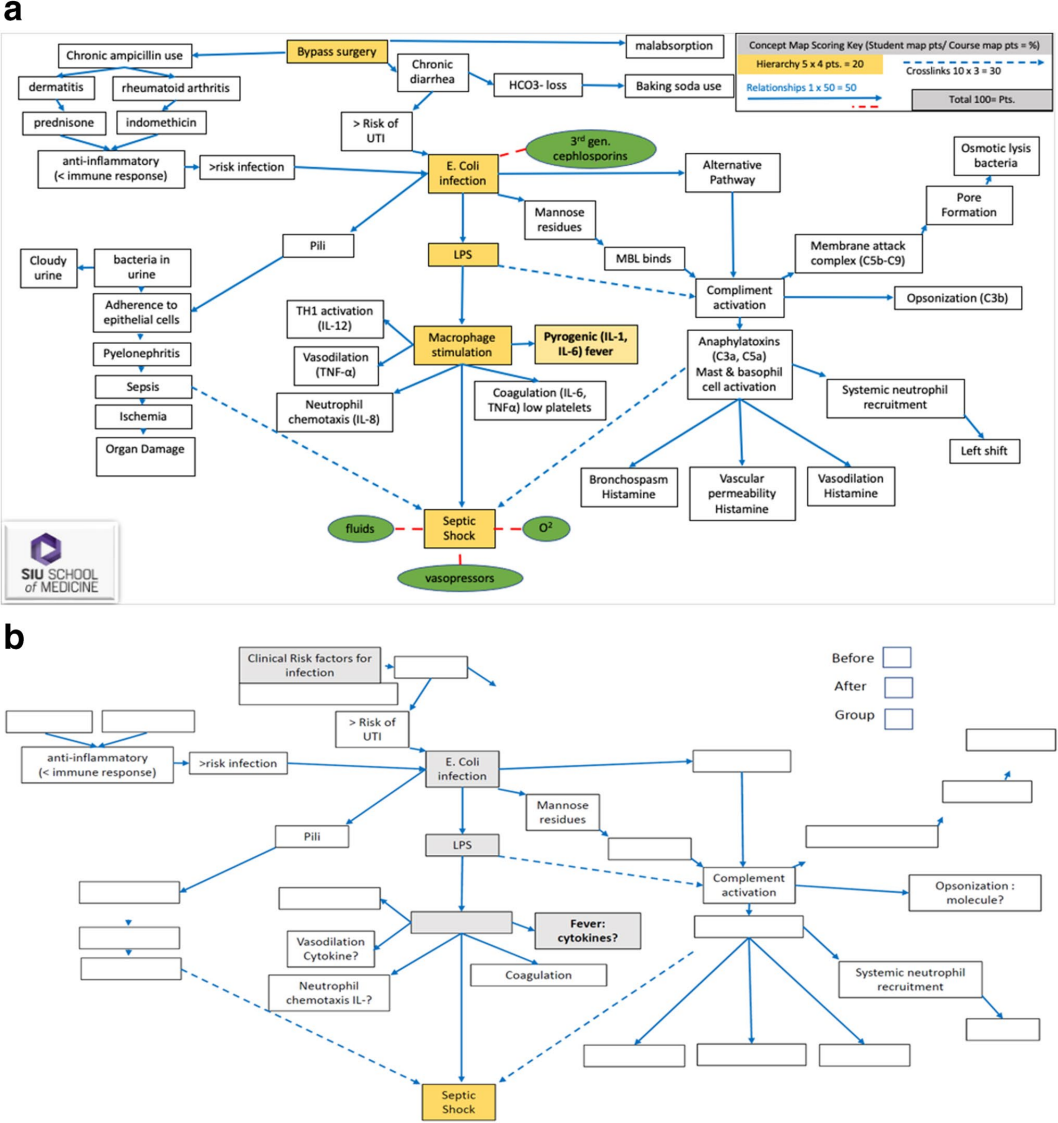
**a** Concept map (scoring algorithm): The legend for scoring the algorithm is located in the upper right region of the map. Answered hierarchical cellular connections (yellow/gray boxes) 4 points; relationships (blue solid line), 1 point; cross-linking cellular concepts (blue dotted line), 3 points. Total points possible 100 points. **b** Concept map provided to the students before and after computer simulation

Figure 3A illustrates the specific criteria for scoring the concept maps, emphasizing the three core components: hierarchy, cross-links, and relationships. Hierarchical connections, indicated by yellow/gray boxes, are awarded 4 points for each correct connection. Relationships, represented by blue solid lines, are given 1 point per accurate association. Cross-links between concepts, represented by blue dotted lines, are awarded 3 points for each valid connection. The total possible score for the CM activity is 100 points. Figure 3B displays representative concept maps completed by students, showing their progress from initial (pre-simulation) to final (post-simulation) understanding of the subject matter. These maps provide a comparative view of how students’ conceptualization and application of the material evolved following the simulation activity, offering insight into their improved ability to integrate and organize complex medical knowledge.

### Statistical Analysis

The statistical analysis of pre- and post-simulation CM scores was conducted using a mixed-model analysis of variance (ANOVA), with the primary outcome being the change in CM scores from pre- to post-intervention. Secondary outcomes included examining the relationship between CM scores and self-assessment scores, as well as assessing long-term knowledge retention, which was evaluated in the third-year student cohort.

To further investigate, a linear mixed model was created to predict CM scores based on group assignment (Group A, B, C, or control) and time (pre- vs. post-simulation), with player ID controlled for across time points. This model analyzed 143 observations from the participants. Estimated marginal means were used to present contrasts, showing differences between groups, between time points, and for group-time interactions. Degrees of freedom were calculated using the Kenward-Roger method, and *p*-values were adjusted for multiple comparisons using the Benjamini and Yekutieli correction. This statistical approach guaranteed interpretation of group and time effects, while controlling for potential biases. *p*-values less than 0.05 were considered statistically significant.

## Results

1. Pre- and post-simulation questionnaires

Learners completed both pre- and post-simulation questionnaires, in which they self-identified knowledge gaps in three key areas (immunology, microbiology, and pathology) (Table 1). A total of 52 responses were collected. Using a 5-point Likert scale (1 = strongly disagree; 5 = strongly agree), students rated their readiness and knowledge of sepsis-related disease processes. An initial review of the surveys did not reveal notable differences between pre- and post-simulation responses. However, questions 15, 16, and 17, which focused on awareness of knowledge gaps in the three specified areas, showed a modest increase following the simulation. The average rating for students’ pre-simulation self-assessment ranged between 3.1 and 3.5, while post-simulation ratings increased to approximately 3.9. Despite these changes, statistical analysis did not demonstrate a significant difference for these questions or any others on the survey. This suggests that while the computer-simulated exercise may have increased students’ perceived awareness of their knowledge gaps, it did not result in a measurable impact on their self-reported readiness or understanding of sepsis-related concepts.

**Table 1:** Pre- and post-simulation self-assessment questionnaires**.** Data are presented as the average response for the three groups. Data for survey questions 15, 16, and 17 are included. **Pre** data correspond to the average of the students in that group before the interactive 3D courseware. **Post** data correspond to the average response of the students in that group on the specific survey question after the interactive 3D courseware. Group A completed the tasks at the start of the sepsis case in the curriculum, Group B at the midpoint, and Group C at the end, with no breaks between tasks for any group

Group	nPre	nPost	Pre^1^	Post^1^	Paired Wilcoxon	*p*^2^
Q15: I am aware of my knowledge GAPs in Immunology						
A	19	19	3 (2, 4)	4 (3, 4)	1.5	0.134
B	18	17	4 (3, 4)	4 (3, 4)	3.0	0.233
C	15	15	4 (3, 4)	4 (2.5, 4)	4.0	0.789
Q16: I am aware of my knowledge GAPs in Microbiology						
A	19	19	4 (2.5, 4)	4 (4, 4)	5	0.0749
B	18	17	4 (4, 4)	4 (4, 4)	15	0.3900
C	15	15	4 (4, 4)	4 (4, 4)	7	0.5200
Q17: I am aware of my knowledge GAPs in Pathology						
A	19	19	3 (2, 4)	4 (4, 4)	10.0	0.0118
B	18	17	3 (2, 4)	4 (3, 4)	15.5	0.0640
C	15	15	3 (2, 4)	4 (2, 4)	22.5	0.6430

^1^Median (IQR)

^2^*p*-values adjusted based on Benjamini and Yekutieli (2001)

2. Learner feedback on the interactive 3D courseware (computer simulation)

At the end of the post-simulation questionnaire, learners had the opportunity to provide feedback on the computer simulation, yielding over 50 individual responses (Table 2). Many students praised the simulation’s usefulness, while 46 offered constructive suggestions for improvement. Two key points emerged from the feedback: learners requested a more readable background color for the simulation questions and expressed a desire for immediate feedback on the correctness of their responses. Notably, 13 students specifically requested better feedback rationale explaining why their answers were right or wrong. While the self-assessment survey did not indicate a significant change in learners’ self-assessment due to the simulation (Table 1), the students’ suggestions highlighted areas for enhancement.

**Table 2:** Results of feedback of responses by group from 52 learners. Group A completed the tasks at the start of the sepsis case in the curriculum, Group B at the midpoint, and Group C at the end, with no breaks between tasks for any group

	Group A	Group B	Group C
Positive feedback	Useful Learning Tool (3) Game metrics (health bar, cost/length of stay) provided a positive experience Keeps attention and solidifies knowledge (1)	Enjoyed Concept (3) Game metrics (health bar, cost/length of stay) provided a positive experience (1)	
	“It’s a great idea. Gamification helps to keep attention and cement things in a fun way”	“Overall cool concept”	
Constructive feedback	Higher clarity of text/images/questions on the software; some questions were hard to read or confusing (8) More clarity if answer was correct or incorrect (5) More explanations of incorrect answers (2) Game would be better utilized as a review tool rather than initial exposure (1) Add H&P Questions (1)	Higher clarity of text/images/questions on the software; some questions were hard to read or confusing (7) More clarity if answer was correct or incorrect (5) Desired more features for gamification (3)	Higher clarity of text/images/questions on the software; some questions were hard to read or confusing (4) More clarity if answer was correct or incorrect (3) Desired more features for gamification (1) More clarity of patient’s clinical picture (1)
	“Sometimes the font was kind of hard to read but besides that, everything else was perfect” “[The game] was seemingly more suited to people who have studied the topic and want to review, wouldn’t recommend for first time seeing material”	“The diagrams could also be a little more clear to read if they were enlarged or had a zoom feature. Great job!” “I would have liked the game to be more interactive than just questions and answers”	“At times, [I] was unsure if I was selecting the correct answer” “Include a general synopsis of patient background and clear points/facts about patient’s treatment.”

3. Concept mapping

### Pre-intervention

The initial analysis of the CM data focused on learners’ performance on the pre-intervention CM. All three intervention groups demonstrated significant improvements compared to the control group (those completing the CM without exposure to the sepsis case). Students in Group A, who completed the CM early in the sepsis case within the medical curriculum, scored an average of 8.74 (SE = 2.90) points higher than the control group (*n* = 19, *p* = 0.021). Group B, who completed the CM midway through the case, scored an average of 10.07 (SE = 2.94) points higher (*n* = 18, *p* = 0.008). Group C, who completed the CM at the end of the case, outperformed the control group by 14.47 (SE = 3.09) points on average (*n* = 15, *p* < 0.001) (Table 3). These findings suggest that engaging with the scaffolded CM, even prior to the simulation, significantly enhanced students’ ability to integrate and organize their knowledge, with greater improvements seen as students progressed through the case.

**Table 3:** Comparison of individual pre-simulation concept map scores. Group A completed the tasks at the start of the sepsis case in the curriculum, Group B at the midpoint, and Group C at the end. A control group (*n* = 15) consisted of students who completed only the pre-simulation CM without exposure to the sepsis case (Year 1 medical students with a foundation in immunology)

Group	Mean 1	Mean 2	Difference	SE	*p*-value
A—Control	12	3.26	8.74	2.90	0.021
B—Control	13.33	3.26	10.07	2.94	0.008
B—A	13.33	12	1.33	2.94	1
C—Control	17.73	3.23	14.47	3.09	< 0.001
C—A	17.73	12	5.73	3.09	0.324
C—B	17.73	13.33	4.40	3.13	0.684

Degrees of freedom method: Kenward-Roger

Confidence level used: 0.95

*p*-values adjusted based on Benjamini and Yekutieli (2001)

### Post-intervention

Post-intervention CM scores were compared across all groups (Table 4). The difference between Group A and Group B scores was minimal, only 0.32 (SE = 2.95) points (*p* = 1.0). However, Group C, which completed the simulation at the end of the sepsis case within the medical curriculum, scored significantly higher than both Group A and Group B, with an average increase of 9.6 (SE = 3.09) points over Group A (*p* = 0.018) and 9.3 (SE = 3.14) points over Group B (*n* = 15, *p* = 0.023). Third-year medical students, who had previously participated in the simulation, scored 15 (SE = 2.83) points higher than Group A (*p* < 0.001) and 14.7 (SE = 2.89) points over Group B (*p* < 0.001), but their scores were not significantly higher than those of Group C, with a difference of only 5.42 (SE = 3.03) points (*p* = 0.344). This suggests that Group C, by completing the simulation later in the learning process, achieved greater knowledge integration, similar to the third-year students. Additionally, the retained knowledge demonstrated by third-year students further indicates the long-term benefits of the simulation activity.

**Table 4:** Comparison of individual post-simulation concept map scores. Group A completed the tasks at the start of the sepsis case in the curriculum, Group B at the midpoint, and Group C at the end. A group of third-year medical students (*n* = 10), who had completed the full activity the previous year, was included to assess knowledge retention

Group	Mean 1	Mean 2	Difference	SE	*p*-value
B—A	17.59	17.26	0.32	2.95	1
C—A	26.87	17.26	9.60	3.09	0.018
C—B	26.87	17.59	9.28	3.14	0.023
3er Yr.—A	32.29	17.26	15.02	2.83	< 0.001
3er Yr.—B	32.29	17.59	14.7	2.89	< 0.001
3er Yr.—C	32.29	26.87	5.42	3.03	0.344

Degrees of freedom method: Kenward-Roger

Confidence level used: 0.95

*p*-values adjusted based on Benjamini and Yekutieli (2001)

### Overall CM Improvement

We compared the changes in pre- and post-intervention scores across the individual groups (Table 5). Group A experienced an increase of 5.26 (SE = 0.98) points from pre- to post-intervention session (*n* = 19, *p* < 0.001). Group B saw an increase of over 4.25 (SE = 1.03) points (*n* = 17, *p* = 0.002). In contrast, Group C demonstrated a more substantial improvement, with an increase of 9.13 (SE = 1.10) points (*n* = 15, *p* < 0.001). Notably, the score increase in Group C was more than double that of Group B and nearly twice that of Group A.

**Table 5:** Change in concept map scores pre-simulation session versus post-simulation session for all individuals in each group

Group	Mean 1	Mean 2	Difference	SE	*p*-value
A	17.26	12	5.26	0.98	< 0.001
B	17.59	13.33	4.25	1.03	0.002
C	26.87	17.73	9.13	1.10	< 0.001

Degrees of freedom method: Kenward-Roger

Confidence level used: 0.95

*p*-values adjusted based on Benjamini and Yekutieli (2001)

Finally, we compared the changes in pre- to post-CM scores among the groups (Table 6). The difference in score changes between Group B and Group A was − 1.01 (SE = 1.43), which was not statistically significant. Similarly, the difference between Group C and Group A was 3.87 (SE = 1.51), also not statistically significant. In contrast, the increase in CM scores for Group C was significantly different from that of Group B, with a difference of 4.88 (SE = 1.51) (*p* < 0.018).

**Table 6:** Comparison of the change (pre-post concept) between groups

Group	Mean 1	Mean 2	Difference	SE	*p*-value
A	4.25	5.26	− 1.01	1.43	1
B	9.13	5.26	3.87	1.48	0.061
C	9.13	4.25	4.88	1.51	0.018

Degrees of freedom method: Kenward-Roger

Confidence level used: 0.95

*p*-values adjusted based on Benjamini and Yekutieli (2001)

## Discussion

### Pre- and Post-intervention Self-assessment

The results from the pre- and post-simulation questionnaires provide valuable insights into learners’ self-assessment of their knowledge gaps in immunology, microbiology, and pathology, particularly in the context of sepsis-related disease processes. Initial analyses revealed no notable differences between pre- and post-simulation responses, indicating that the simulation may not have significantly altered students’ overall self-reported readiness or comprehension of sepsis-related concepts. This finding aligns with existing literature suggesting that self-assessments can sometimes yield limited insights into actual learning gains [28–30]. However, it is noteworthy that questions 15, 16, and 17, which focused specifically on learners’ awareness of their knowledge gaps, did exhibit a modest increase post-simulation. This suggests that the simulation may have effectively heightened students’ awareness of their deficiencies in understanding critical concepts.

The increase in awareness, albeit not statistically significant, implies that students may benefit from reflective exercises that prompt them to consider their learning needs more critically. Despite the absence of statistically significant changes in self-reported readiness and understanding, the modest improvements in awareness point to the potential of interactive 3D courseware as tools for nurturing metacognitive skills. By motivating students to reflect on their knowledge gaps, simulations may encourage a more proactive approach to their learning, even if this does not immediately translate into enhanced self-assessment scores. The lack of significant findings raises important considerations for the design and implementation of educational interventions. While simulations can enhance engagement and foster awareness, they may need to be supplemented with additional instructional strategies to effectively translate increased awareness into concrete learning outcomes [31, 32]. This could involve integrating formative assessments, guided discussions, or additional resources that address identified knowledge gaps directly.

These results can lead to further discussions on metacognition. Metacognition in medical education refers to the process by which students become aware of and regulate their own learning. It involves reflecting on one’s knowledge, identifying gaps, and adapting learning strategies accordingly [33]. In the context of medical education, promoting metacognitive skills is crucial for students to integrate complex concepts, such as those in immunology, microbiology, and pathology, into their clinical practice. Tools like self-assessments, reflective exercises, and CM can help students develop these skills by encouraging them to actively engage with the material, monitor their understanding, and make adjustments to enhance their learning.

To summarize this first approach, while the computer simulation exercise did not produce significant changes in self-reported readiness or understanding, it appears to have contributed to increased awareness of knowledge gaps among students. Future research should explore the long-term impact of such awareness on learning outcomes and consider how simulations can be better integrated with other educational methods to maximize their effectiveness in promoting deeper understanding of complex subjects like sepsis.

### Approach to Measuring the Impact of Computer Simulation on Concept Map Learning in the Curriculum

In contrast to the results of the student survey self-assessments, our findings indicate that the interactive 3D courseware (computer simulation) significantly enhances learners’ ability to complete CM. Notably, even without the simulation, the current curriculum alone contributed to improvements in CM abilities. For instance, Group A learners, who engaged with the material early in the case, produced pre-simulation CM scores higher than those of the control group (Table 3). This suggests that prior exposure to the curriculum effectively facilitated linkages and conceptual integration, even at the outset of the case. Pre-simulation CM scores showed significant improvement in all three groups compared to the control group showing the ability of MS2s to establish stronger baseline CM, likely due to their advanced coursework. Interestingly, while third-year learners’ CM scores were only 5.62 points higher than those of Group C learners, the latter, who completed their assessments later in the case, demonstrated significantly higher scores than both the mid-case (Group B) and early-case (Group A) learners (Table 4). This observation has two important implications. First, it indicates that the knowledge acquired by the end of the case nearly matches that of third-year medical students, as measured by CM performance. The comparable scores suggest that understanding of cellular mechanisms learned during the case is retained over time, as evidenced by the third-year students’ scores remaining stable one-year post-computer simulation.

To further explore the interplay between case instruction and computer simulation, we assessed the CM skills of learners both before and immediately after the simulation for each group (Table 5). All groups exhibited improvements in post-simulation scores compared to their pre-simulation results. One could argue that these increases can be attributed to the interactive 3D courseware (computer simulation), given that measurements were taken immediately following the intervention.

Finally, we investigated the optimal timing for the computer simulation within the case to maximize its impact on learning the cellular basis of disease (Table 6). The pre-to-post changes in CM scores between early-case learners (Group A) and mid-case learners (Group B) were not significantly different. However, a significant increase was observed when comparing the pre- and post-simulation scores of Group C with those of Group B. This finding suggests that placing the computer simulation at the end of the case may yield the greatest educational benefit. It is plausible that the simulation allows students to better assimilate the various elements of the case into a cohesive CM, reinforcing their understanding of the material.

Our findings align with previous research suggesting that simulation-based education is a powerful tool for enhancing learning in medical education [34–36]. The 3D simulation platform provided an immersive environment where students could explore the cellular processes underlying sepsis and link this knowledge to patient outcomes. By engaging with the simulation, students were able to visualize complex pathophysiological mechanisms, making abstract concepts more tangible and clinically relevant. CM complemented this experience by offering a structured approach to reinforce and assess students’ understanding of these processes. Rather than serving as the primary intervention, CM functioned as a cognitive scaffolding tool, helping students integrate new information from the simulation with their prior knowledge. The combination of simulation and CM encouraged deeper engagement by requiring students to actively organize and synthesize information rather than passively absorb it. Additionally, CM allowed for the identification of misconceptions or gaps in understanding that might not have been evident through the simulation alone. The integration of these two tools—an interactive simulation to provide experiential learning and CM to facilitate cognitive organization—offers a promising strategy for teaching complex medical concepts. This approach aligns with contemporary educational frameworks that emphasize multimodal learning, where complementary instructional strategies enhance comprehension and long-term retention. While the simulation was the central learning modality in our study, CM played a supportive yet essential role in reinforcing conceptual connections, ultimately enhancing the overall effectiveness of the educational intervention.

In addition, as a valuable part of assessment, CM offers several advantages over traditional multiple-choice question (MCQ) assessments in evaluating conceptual understanding. Unlike MCQs, which primarily test recall and recognition of discrete facts, CM requires students to actively construct and organize knowledge, promoting deeper comprehension and long-term retention. By visually mapping relationships between key concepts, CM facilitates the integration of new information with prior knowledge, allowing for a more holistic representation of understanding. Additionally, CM provides insight into students’ reasoning processes, revealing misconceptions or gaps in knowledge that MCQs might not detect [37]. In the context of our study, CM served as both a learning and assessment tool, enabling students to synthesize information from the 3D simulation and apply it in a structured manner. This approach aligns with constructivist learning theories, which emphasize active engagement and knowledge organization as essential components of meaningful learning. Given these benefits, CM represents a valuable alternative or complement to traditional assessment methods, particularly when evaluating complex medical concepts that require higher-order thinking skills.

### Optimizing the Integration of Interactive 3D Courseware in Case-Based Learning: Insights on Timing and Assessment

Implementing computer-based instructional elements into an existing curriculum requires careful instructional decision-making and consideration of multiple factors. Building on previous work exploring strategies for integrating interactive 3D courseware into case-based curricula, we summarize key insights as follows:

- Interactive 3D courseware can serve as valuable tools for students to self-evaluate their understanding of complex disease mechanisms.
- CM scores may offer a more effective way for students to identify learning gaps compared to traditional self-assessment surveys.
- It is challenging to clearly distinguish the learning contributions of the current curriculum from those of a computer simulation element. Further research is needed to clarify their respective impacts.
- Measuring pre- and post-simulation CM scores at distinct intervals may help differentiate the contributions of interactive 3D courseware from the overall effect of curricular material.

The findings suggest that adding a formative element like a computer simulation may have the greatest impact when introduced toward the end of the case, rather than at the beginning. In this study, students demonstrated retention of disease-related knowledge for up to a year, as measured by CM scores.

One limitation of this study is the relatively small sample size, which may limit the generalizability of the results. Additionally, while student feedback indicated high engagement with the simulation, some students felt that CM and the simulation were not seamlessly integrated. Future studies should focus on refining the integration of these tools and exploring the long-term impact of this educational approach on clinical performance.

In conclusion, our study underscores the value of integrating interactive 3D courseware into medical curricula, as they not only enhance CM learning but also contribute to the retention of knowledge over time. Future research should explore additional strategies for optimizing the timing and implementation of such simulations to further enhance student learning outcomes.

## Data Availability

The datasets generated during and/or analyzed during the current study are available from the corresponding author on reasonable request.

## References

[1] Alotaibi AA, Cordero MAW. Assessing medical students’ knowledge of genetics: basis for improving genetics curriculum for future clinical practice. Adv Med Educ Pract. 2021;12:1521–30. 10.2147/amep.S337756. 35002351 10.2147/AMEP.S337756 PMC8722570

[2] Brynhildsen J, Dahle LO, Fallsberg MB, Rundquist I, Hammar M. Attitudes among students and teachers on vertical integration between clinical medicine and basic science within a problem-based undergraduate medical curriculum. Med Teach. 2002;24:286–8. 10.1080/01421590220134105. 12098415 10.1080/01421590220134105

[3] Kulasegaram K, Rangachari PK. Beyond “formative”: assessments to enrich student learning. Adv Physiol Educ. 2018;42:5–14. 10.1152/advan.00122.2017. 29341810 10.1152/advan.00122.2017

[4] Swan Sein A, Rashid H, Meka J, Amiel J, Pluta W. Twelve tips for embedding assessment for and as learning practices in a programmatic assessment system. Med Teach. 2021;43:300–6. 10.1080/0142159x.2020.1789081. 32658603 10.1080/0142159X.2020.1789081

[5] Michael J. In pursuit of meaningful learning. Adv Physiol Educ. 2001;25:145–88. 11824191 10.1152/advances.2001.25.3.145

[6] Woods NN, Neville AJ, Levinson AJ, Howey EH, Oczkowski WJ, Norman GR. The value of basic science in clinical diagnosis. Acad Med. 2006;81(10 Suppl):S124–7. 10.1097/00001888-200610001-00031. 17001122 10.1097/00001888-200610001-00031

[7] Martin GS, Mannino DM, Eaton S, Moss M. The epidemiology of sepsis in the United States from 1979 through 2000. N Engl J Med. 2003;348:1546–54. 10.1056/NEJMoa022139. 12700374 10.1056/NEJMoa022139

[8] Castellanos-Ortega A, Suberviola B, García-Astudillo LA, et al. Impact of the Surviving Sepsis Campaign protocols on hospital length of stay and mortality in septic shock patients: results of a three-year follow-up quasi-experimental study. Crit Care Med. 2010;38:1036–43. 10.1097/CCM.0b013e3181d455b6. 20154597 10.1097/CCM.0b013e3181d455b6

[9] Novak JD, Gowin DB. Learning how to learn. New York: Cambridge University Press; 1984.

[10] Torre DM, Durning SJ, Daley BJ. Twelve tips for teaching with concept maps in medical education. Med Teach. 2013;35:201–8. 10.3109/0142159X.2013.759644. 23464896 10.3109/0142159X.2013.759644

[11] Daley BJ, Torre DM. Concept maps in medical education: An analytical literature review. Med Educ. 2010;44:440–8. 20374475 10.1111/j.1365-2923.2010.03628.x

[12] Daley BJ, Shaw CR, Balistrieri T, Glasenapp K, Piacentine L. Concept maps: A strategy to teach and evaluate critical thinking. J Nurs Educ. 1999;38:42–7. 9921788 10.3928/0148-4834-19990101-12

[13] West DC, Pomeroy JR, Park JK, Gerstenberger EA, Sandoval J. Critical thinking in graduate medical education – A role for concept mapping assessment? J Am Med Assoc. 2000;284:1105–10. 10.1001/jama.284.9.1105 10974689

[14] Chang C-Y, Yang JC. Concept mapping in computer-supported learning environments: A bibliometric analysis. Interact Learn Environ. 2023;31:6678–95.

[15] Hartmeyer R, Stevenson MP, Bentsen P. A systematic review of concept mapping-based formative assessment processes in primary and secondary science education. Assessment Educ: Princ Policy Pract. 2018;25:598–619.

[16] Machado CT, Carvalho AA. Concept mapping: Benefits and challenges in higher education. J Contin High Educ. 2020;68:38–53.

[17] Chen W, Allen C. Concept mapping: providing assessment of, for, and as learning. Med Sci Educ. 2017;27:149–53.

[18] Torre DM, Daley B, Stark-Schweitzer T, Siddartha S, Petkova J, Ziebert M. A qualitative evaluation of medical student learning with concept maps. Med Teach. 2007;29:949–55. 10.1080/01421590701689506. 18158670 10.1080/01421590701689506

[19] Torre D, Daley BJ, Picho K, Durning SJ. Group concept mapping: An approach to explore group knowledge organization and collaborative learning in senior medical students. Med Teach. 2017;39:1051–6. 10.1080/0142159x.2017.1342030. 28681636 10.1080/0142159X.2017.1342030

[20] Morse D, Jutras F. Implementing concept-based learning in a large undergraduate classroom. CBE Life Sci Educ. 2008;7:243–53. 18519616 10.1187/cbe.07-09-0071 PMC2424300

[21] van der Vleuten CP, Schuwirth LW, Driessen EW, Dijkstra J, Tigelaar D, Baartman LK, van Tartwijk J. A model for programmatic assessment fit for purpose. Med Teach. 2012;34:205–14. 22364452 10.3109/0142159X.2012.652239

[22] Ruiz-Primo MA, Shavelson RJ. Problems and issues in the use of concept maps in science assessment. J Res Sci Teach. 1996;33:569–600.

[23] Kinchin IM, Hay DB, Adams A. How a qualitative approach to concept map analysis can be used to aid learning by illustrating patterns of conceptual development. Educ Res. 2000;42:43–57.

[24] Durkin K. Videogames and young people with developmental disorders. Rev Gen Psychol. 2010;14:122–40.

[25] Graafland M, Schraagen JM, Schijven MP. Systematic review of serious games for medical education and surgical skills training. Br J Surg. 2012;99:1322–30. 10.1002/bjs.8819. 22961509 10.1002/bjs.8819

[26] de Wit-Zuurendonk LD, Oei SG. Serious gaming in women’s health care. BJOG. 2011;118(Suppl 3):17–21. 10.1111/j.1471-0528.2011.03176.x. 10.1111/j.1471-0528.2011.03176.x 22039888

[27] Kato PM. Video games in health care: Closing the gap. Rev Gen Psychol. 2010;14:113–21.

[28] Eva KW, Regehr G. Self-assessment in the health professions: a reformulation and research agenda. Acad Med. 2005;80(10 Suppl):S46-54. 16199457 10.1097/00001888-200510001-00015

[29] Davis DA, Mazmanian PE, Fordis M, van Harrison R, Thorpe KE, Perrier L. Accuracy of physician self-assessment compared with observed measures of competence. JAMA. 2006;296:1094–102. 10.1001/jama.296.9.1094. 16954489 10.1001/jama.296.9.1094

[30] Johnson WR, Durning SJ, Allard RJ, Barelski AM, Artino AR Jr. A scoping review of self-monitoring in graduate medical education. Med Educ. 2023;57:795–806. 36739527 10.1111/medu.15023

[31] Mahan JD, Clinchot D. Why medical education is being (inexorably) re-imagined and re-designed. Curr Probl Pediatr Adolesc Health Care. 2014;44:137–40. 24981662 10.1016/j.cppeds.2014.01.002

[32] Rohlfsen CJ, Sayles H, Moore GF, Mikuls TR, O’Dell JR, McBrien S, Johnson T, Fowler ZD, Cannella AC. Innovation in early medical education, no bells or whistles required. BMC Med Educ. 2020;20(1):39. 32033553 10.1186/s12909-020-1947-6 PMC7006170

[33] Gonullu I, Artar M. Metacognition in medical education. Educ Health. 2014;27:225–6. 10.4103/1357-6283.143784. 10.4103/1357-6283.143784 25420992

[34] Choy CL, Liaw SY, Goh EL, See KC, Chua WL. Impact of sepsis education for healthcare professionals and students on learning and patient outcomes: a systematic review. J Hosp Infect. 2022;122:84–95. 35045340 10.1016/j.jhin.2022.01.004

[35] Fernández-Ros N, Alegre F, Huerta A, et al. Acquiring sepsis competencies through simulation-based learning bundle during intermediate care unit internship. Medicine (Baltimore). 2021;100:e24483. 10.1097/md.0000000000024483. 33592900 10.1097/MD.0000000000024483 PMC7870168

[36] Ribeiro C, Monteiro M, Hauge JB, Pereira J, Antunes T. Sepsis fast track: A simulation game for clinical education based on the sepsis fast track protocol. IEEE; 2016:1–8.

[37] Masood Z, Jawaid M, Aly SM, Muhammad Z. Comparison of Surgery test scores using Concept Maps and Interactive Lectures among the Undergraduate Medical Students. Pak J Med Sci. 2024;40:2305–8. 10.12669/pjms.40.10.10526. 39554680 10.12669/pjms.40.10.10526 PMC11568694

